# Supplementary material for: Density of states prediction for materials discovery via contrastive learning from probabilistic embeddings
Source: Nat Commun. 2022 Feb 17;13:949. doi: 10.1038/s41467-022-28543-x (PMC8854636; doi:10.1038/s41467-022-28543-x)
Supplement: Supplementary file 1 — Supplementary Info [file 41467_2022_28543_MOESM1_ESM.pdf]

Supplementary Information

Density of States Prediction for Materials Discovery via Contrastive Learning from Probabilistic Embeddings

J. Gregoire et al.

Supplementary Figures

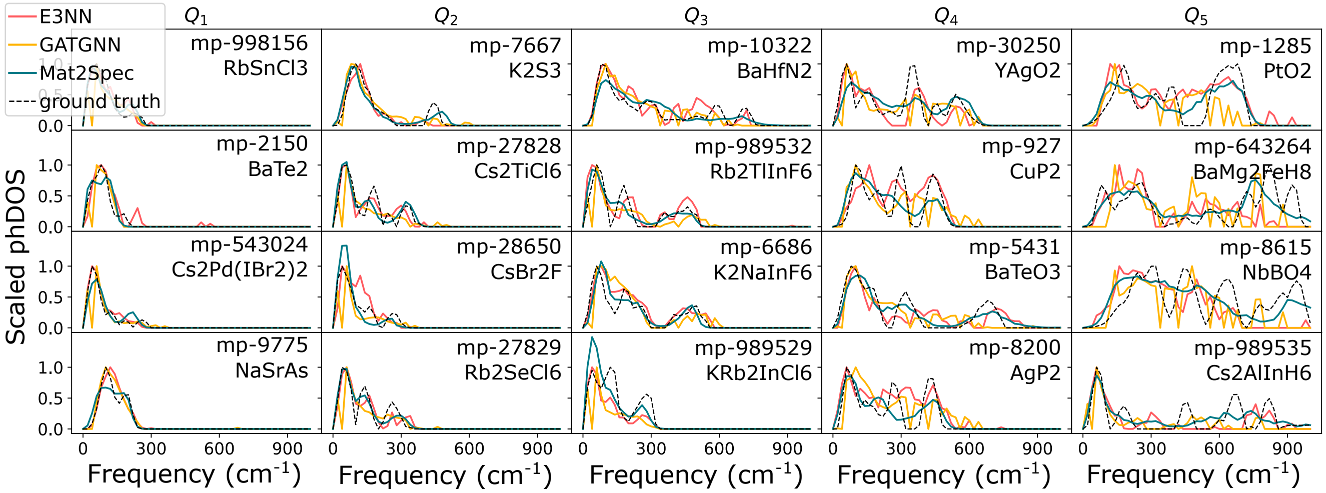

Supplementary Figure 1. **Example phDOS predictions**: For E3NN and GATGNN, the lowest MAE for phDOS prediction occurs using the MaxNorm scaling with MSE training loss. For Mat2Spec, the lowest MAE results from SumNorm scaling and KL training loss. Using these respective settings, ground truth and predicted phDOS is shown for 15 example materials. The 5 columns represent the 5 quintiles from low MAE loss (Q_1, left) to high MAE loss (Q_5, right). In each column, 4 randomly chosen materials are shown. From left to right, the ground truth phDOS is increasingly complex, enhancing the differentiation among the different prediction models. For example, in Q_4 each phDOS has 3 or 4 primary peaks, and Mat2Spec is the only ML model with consistent qualitative accuracy, which leads to the better quantitative metrics of Table 1, especially for the WD loss metric that characterizes the accuracy of the shape of each spectrum.

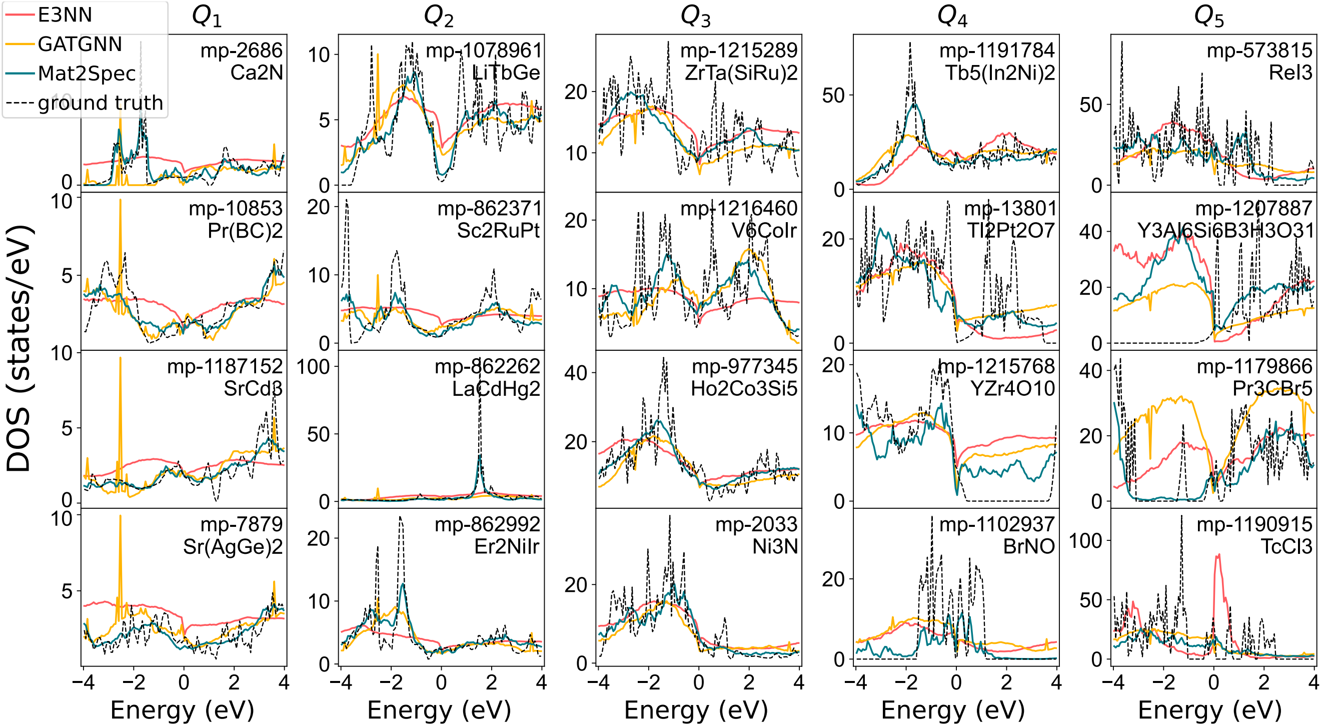

Supplementary Figure 2. Example eDOS predictions: For each ML model, the SumNorm scaling with KL loss provides the best predictions with respect to most of the loss metrics. Using this setting, the ground truth and predicted eDOS is shown for 15 example materials. The 5 columns represent the 5 quintiles from low MAE loss (Q_1, left) to high MAE loss (Q_5, right). In each column, 4 randomly chosen materials are shown. Unlike phDOS, there is no systematic change in the shape of the eDOS across the quintiles, although the materials with higher MAE loss exhibit a higher occurrence of sharp peaks. Mat2Spec consistently predicts a smoothed version of the ground truth, recovering the general shape of the eDOS, which is commensurate with its superior WD loss in Table 2.

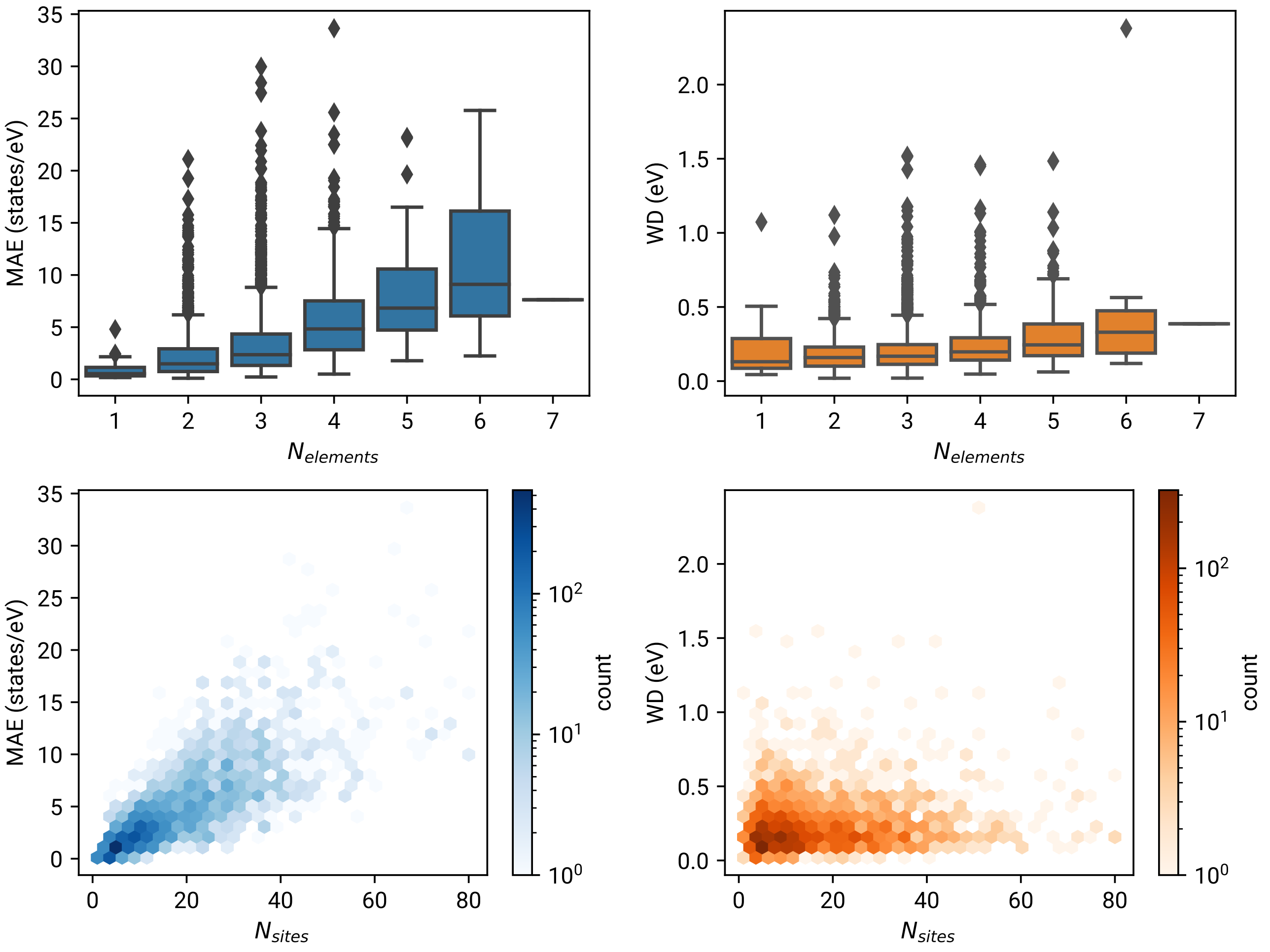

Supplementary Figure 3. Mat2Spec loss vs. material complexity: Each panel contains a point for each test set material using the Mat2Spec SumNorm-KL setting. The prediction loss is plotted as a function of 2 descriptors for the complexity of a material, (top) number of elements and (bottom) number of sites in the unit cell for each material. The expected positive correlation is observed between material complexity and prediction loss. For either the number of elements or the number of sites, the available training data with a similar number of elements or sites also decreases as these numbers increase. Having few training examples of materials with similar complexity exacerbates the challenge of predicting the properties of complex materials. These results inform the trustworthiness of predictions for new materials based on these simple descriptors.

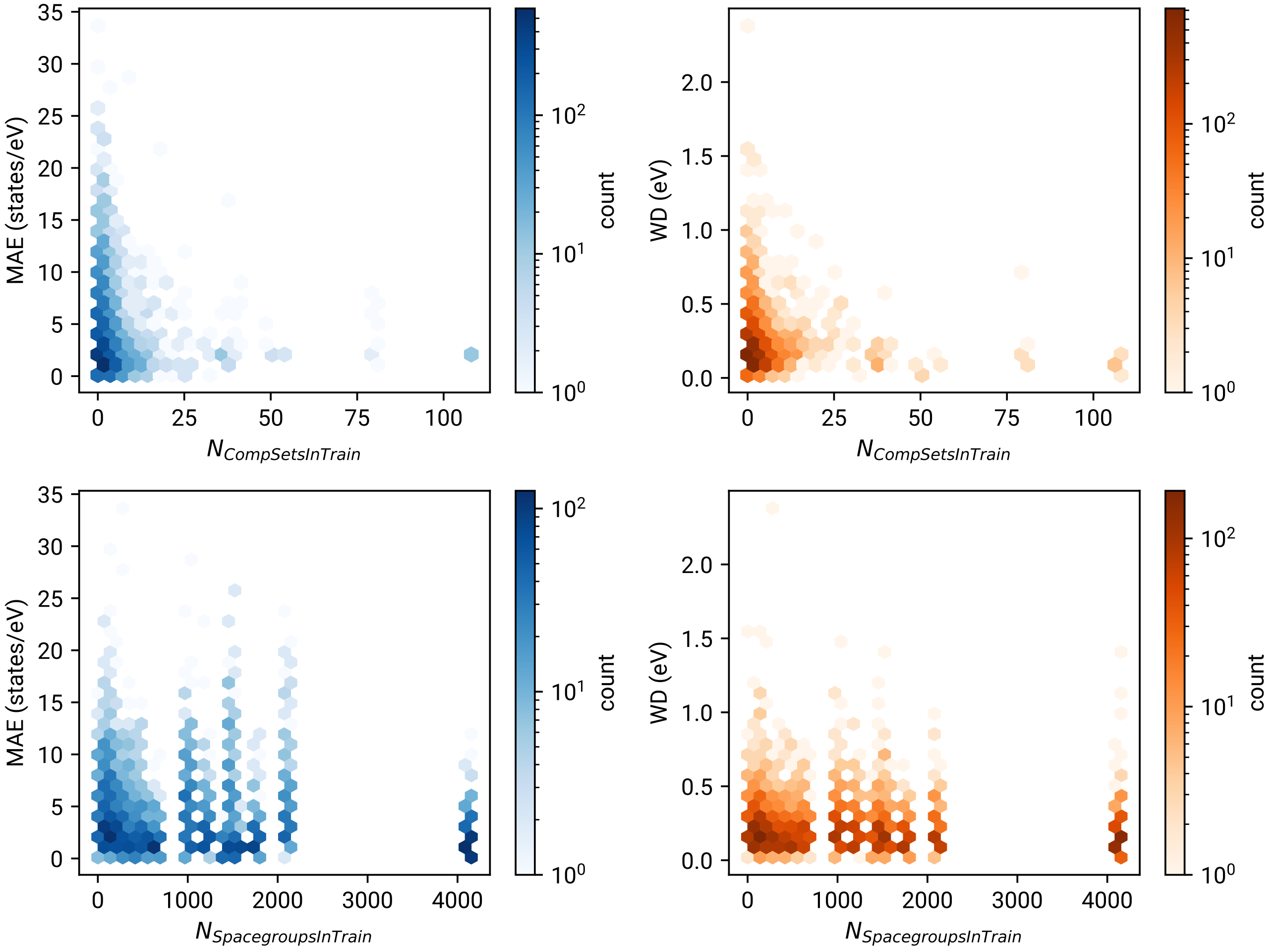

Supplementary Figure 4. Mat2Spec loss vs. similarity of training data: Each panel contains a point for each test set material using the Mat2Spec SumNorm-KL setting. The prediction loss is plotted as a function of 2 descriptors for the similarity to materials in the training data, the number of materials in the training set that have (top) the same set of elements and (bottom) the same spacegroup for each material in the test set. The expected negative correlation is observed between the amount of similar training data and the prediction loss. The relationship is more profound for the number of training examples with the same elements, suggesting that training examples with the same elements in different compositions and/or structures facilitates the model's learning of the chemistry of those elements. These results inform the trustworthiness of predictions for new materials based on these simple descriptors.

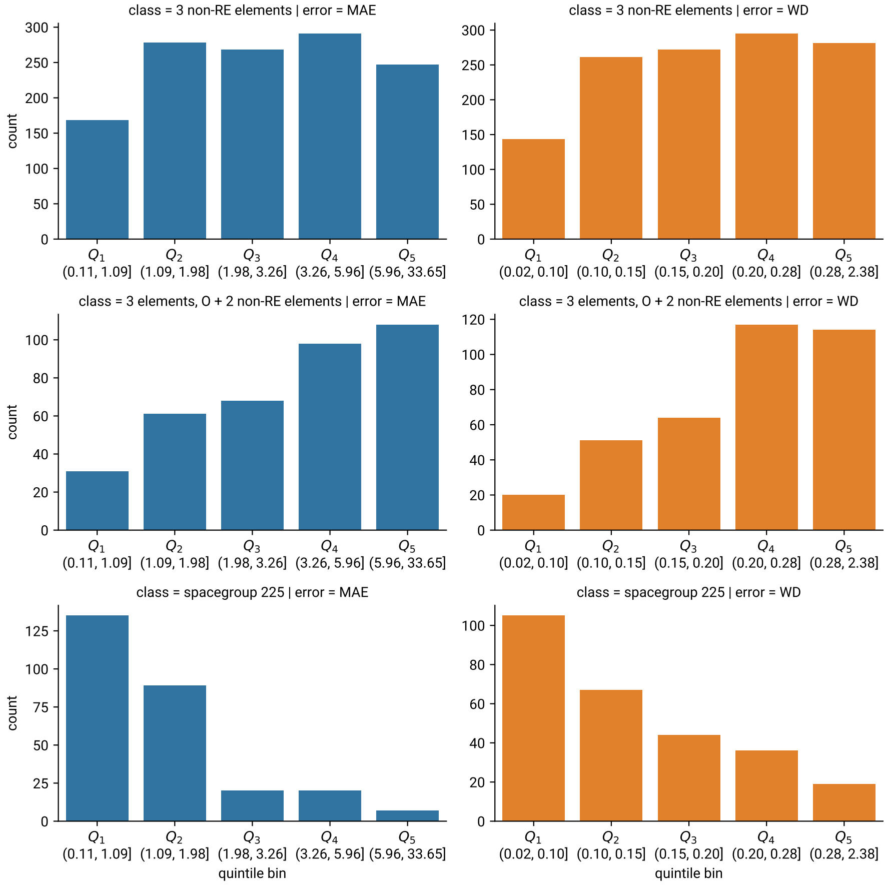

Supplementary Figure 5. Variation in loss among subclasses: Since many materials discovery projects are focused on a particular subclass of materials, the variation in performance of Mat2Spec among different subclasses is shown. The 3 example subclasses are (top) materials containing 3 non-rare-earth elements, (middle) the subset of those that contain oxygen, and (bottom) materials with spacegroup 225. For each subclass, the distribution of losses for test set materials is shown with respect to the MAE and WD quintiles of Mat2Spec SumNorm-KL predictions (the quintiles of the full test set, whose ranges are noted on each horizontal axis). A subclass with the same distribution of losses as the full test set will have equal counts in each quintile. The first subclass has a fairly uniform distribution of loss, while the subset of this subclass for oxygen-containing materials has a distribution substantially skewed toward higher-loss quintiles. Commensurate with the observation from Supplementary Figure 4, where having many training examples with the same spacegroup leads to lower prediction loss, the distribution of loss for the subclass of materials with the most populous spacegroup (spacegroup 225) is strongly skewed toward the lower quintiles. Further study is required to elucidate whether the descriptors in Supplementary Figure 3 and Supplementary Figure 4 explain the distribution of loss within a given subclass, or whether it is the complexity of the materials chemistry with the subclass that underlies the difficulty of the prediction task. The latter appears to be true for ternary oxides.

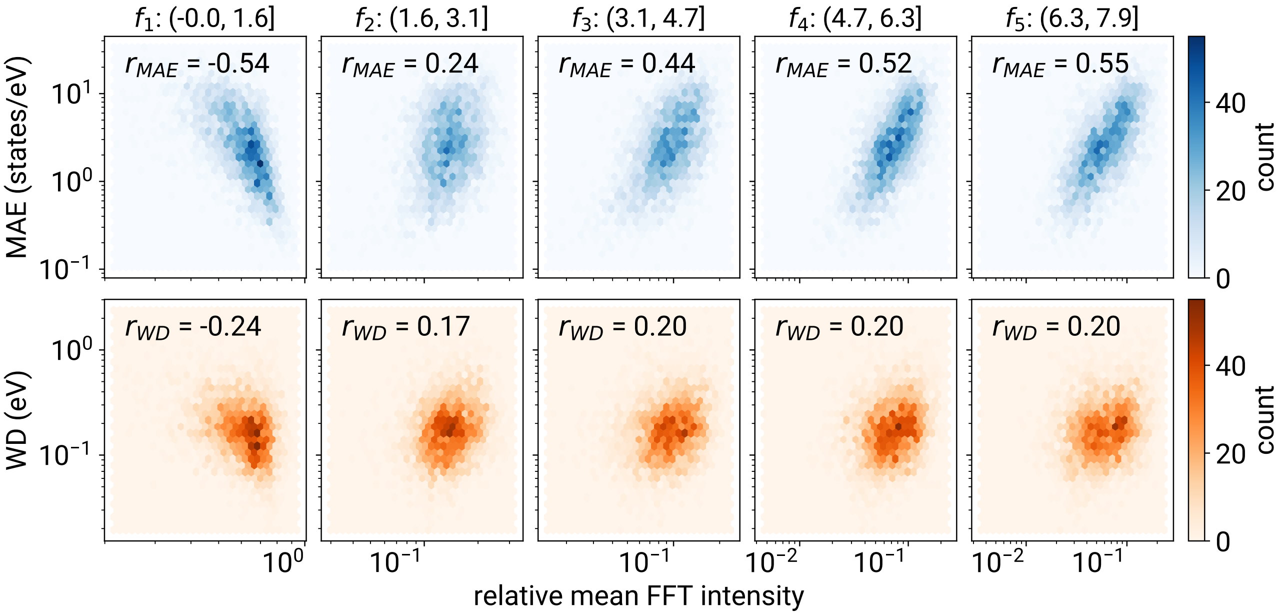

Supplementary Figure 6. Mat2Spec loss vs. Fourier components: The primary observation from inspection of the quintile plots of MAE loss for Mat2Spec SumNorm-KL is that sharp peaks and other high-frequency features are sometimes poorly predicted. To study the generality of this observation, the Fourier transform of each test set eDOS was calculated and divided into 5 frequency ranges. The relative intensity in each of these frequency ranges characterizes whether a given material has primarily low, high, etc. features. The plotted loss in each panel is the (same) prediction loss for the test set materials, and the Pearson correlation coefficient (r) is shown in each panel. For both loss metrics, a moderate negative correlation is observed for the lowest frequency range, and moderate positive correlation is observed for each of the higher frequency ranges. The high level observation is that while Mat2Spec can accurately predict eDOS with high frequency features for some materials, the existence of high frequency features does predispose materials to having higher prediction loss.

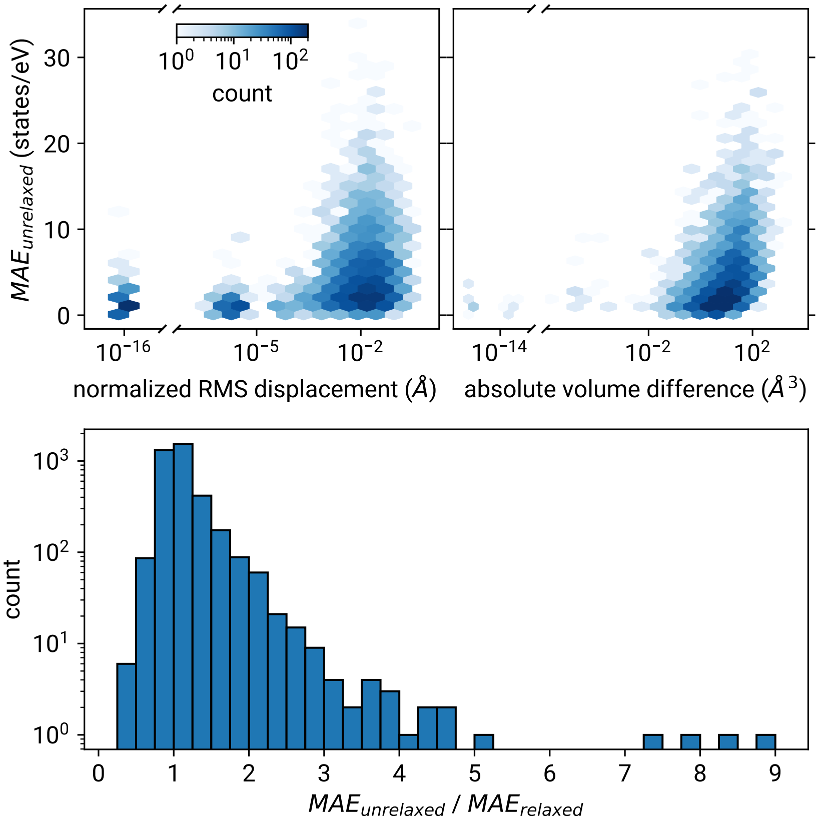

Supplementary Figure 7. Predicting eDOS of unrelaxed structures: The Materials Project makes available the CIF file of each mp-id prior to the DFT-based relaxation of cell parameters and atomic coordinates. While there are multiple sources of the original "unrelaxed" structures, this provides a non-arbitrary example of an unrelaxed structure for each mp-id. The eDOS of the unrelaxed structure is not available (never calculated because the typical workflow is DFT relaxation followed by DFT for eDOS). However, the unrelaxed structures can provide some insight in the ability to predict eDOS without DFT-based cell relaxation. Using the test set, the unrelaxed CIF was provided to the trained Mat2Spec SumNorm-KL model to predict the eDOS, with loss calculated using the DFT ground truth of the respective relaxed structure. The results are shown in the top figures as a function of the extent of structure relaxation, (left) the root mean squared (RMS) difference in atomic coordinates and (right) the absolute change unit cell volume. The few points below the break in each horizontal axis correspond to no atomic coordinates or volume relaxation, respectively. As expected, unrelaxed structures that are more different than their relaxed counterparts have a higher propensity for a large MAE. The Pearson correlation coefficients are 0.19 and 0.35 for the RMS distance and volume difference, respectively. To assess whether the unrelaxed structures could be used as a proxy for the relaxed structure for predicting eDOS, the ratio of MAE for Mat2Spec's prediction using the unrelaxed vs. the relaxed structure was calculated for each test set material, providing the distribution shown at bottom. The mode and median are all near a ratio of 1, and the mean is 1.04, demonstrating that on average using the unrelaxed structures increases the MAE by only 4%. As expected, the distribution does not include any very small values for the MAE ratio, while the distribution extends to high ratios, demonstrating that for some materials using the relaxed structure substantially improves the Mat2Spec prediction. The implications for deploying Mat2Spec on unrelaxed structures is that the propensity of high-MAE outliers may increase compared to starting with relaxed structures, although further analysis of the sensitivity of Mat2Spec prediction with respect to atomic coordinates must be performed, especially for the specific subclass of materials where prediction from unrelaxed structures may be deployed. Overall the results indicate that the utility of Mat2Spec will be amplified by development of generative models with reasonable prediction accuracy for the DFT-relaxed atomic coordinates.

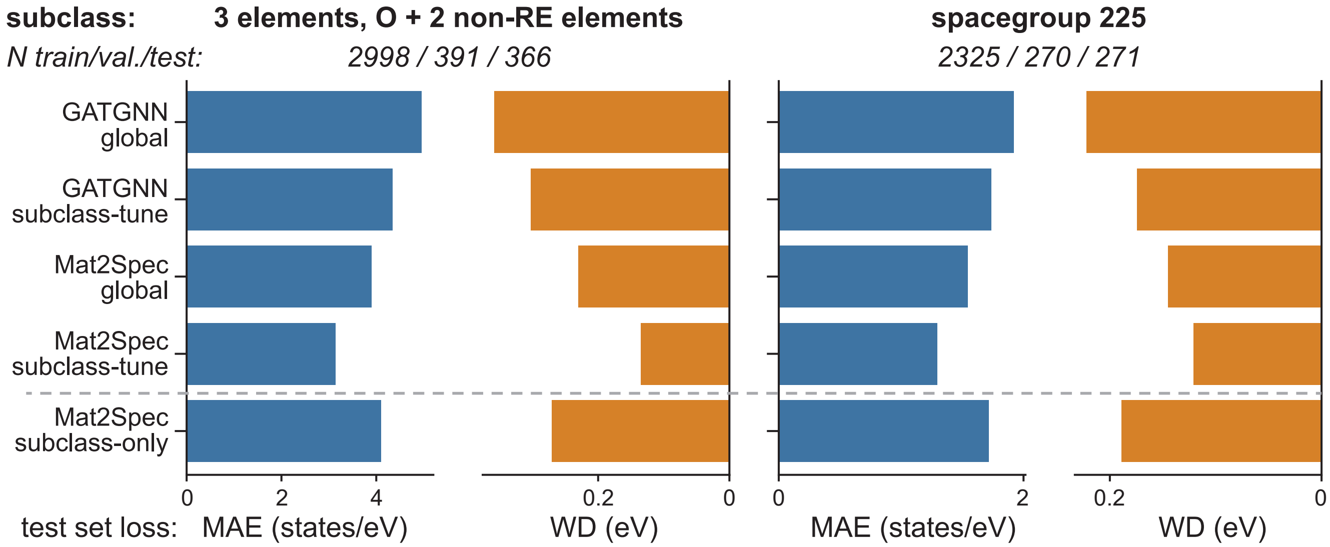

Supplementary Figure 8. Materials subclass eDOS prediction: The two example sub-classes of materials are (left) materials containing 3 elements including oxygen and 2 non-rare-earth elements and (right) materials with spacegroup 225 (see Supplementary Figure 5). These classes were chosen to have 1 subclass defined by composition and the other by structure, while producing subclass datasets of comparable size. The size of the train, validation, and test sets is noted, where each set is taken to be the intersection of the sets from the primary eDOS results and the respective materials subclass. For each subclass, the bars show both the MAE and WD loss for the portion of the original test set that is in the respective sub-class. The results include GATGNN and Mat2Spec in the SumNorm-KL setting with up to 3 different training strategies. The “global” version is trained using the full test set from the primary results in the paper, i.e. not specialized for any specific sub-class, with the loss calculated on the portion of the test set that is within the respective subclass. The “subclass-tune” models are initialized by the respective “global” model with continued training and validation using only the subclass data, which is a type of transfer learning where a model is pre-trained globally and transferred for further training in the sub-domain. For each loss metric and each subclass, Mat2Spec “global” outperforms GATGNN “subclass-tune”, and further improvements are obtained by tuning Mat2Spec for the respective subclass. The improvement from the “subclass-tune” training raises the question of whether comparable performance could be obtained without the transfer learning, which was evaluated by the scenario below the dashed line wherein only the train and validation from the subclass was used to train Mat2Spec from a random initialization. The performance is substantially degraded by removing the transfer learning, although even with only (left) 2998 and (right) 2325 training materials, the “subclass-only” training from Mat2Spec still outperforms all versions of GATGNN, providing 2 specific subclass demonstrations of the observation from Supplementary Figure 8 that Mat2Spec performs well with decreasing training size. The observation that “subclass-only” loss is larger than “global” loss in each case highlights that even when a researcher is focused on a specific materials subclass, training with a broader diversity of materials is better than training locally. Combining these complementary strategies with “subclass-tune” offers additional improvements. Note that for both “subclass-tune” and “subclass-only”, each model was also trained in the Standard-MAE setting to provide the normalization factor, i.e. the same methodology described in the main text.

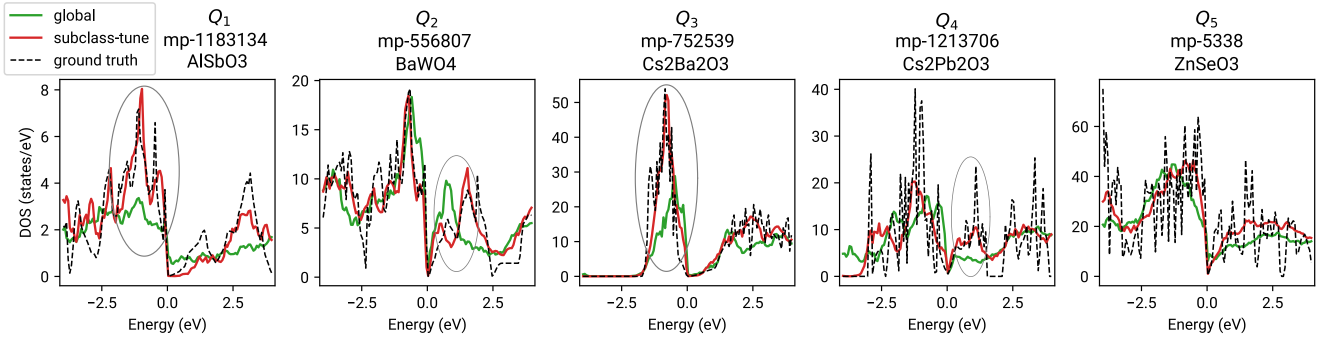

Supplementary Figure 9. Example eDOS subclass refinements: Using the first subclass from Supplementary Figure 8, 3-element materials containing oxygen and 2 non-rare-earth elements, example predictions by Mat2Spec SumNorm-KL are shown for a select example in each MAE quintile. Following the protocol for analogous figures, each mp-id is in the same quintile for the 2 model variants, the "global" model from the main text and the "subclass-tune" model that was tuned on this specific subclass. In Q_1, Q_2, and Q_3 the "global" model correctly predicts the overall shape, with the "subclass-tune" model better capturing specific features. This is somewhat true in the Q_4 example, although for both Q_4 and Q_5, both models appear to predict a smoothed version of the eDOS where the high-level structure is captured but not the many high-frequency features (sharp peaks). The circled regions indicate eDOS features that are better captured by the "subclass-tune" prediction. These examples illustrate the value of the transfer learning strategy described in Supplementary Figure 8, although some aspects of eDOS for some materials remain imperfectly predicted.
